# Mouth-Breathing and the Teeth

**Published:** 1890-03

**Authors:** 


### SELECTIONS.

### Mouth-Breathing and the Teeth.

Dr. Scanes Spicer read a paper at the last meeting of the Odontological Society of London, upon "Nasal Obstruction and Mouth-Breathing as Factors in the Etiology of Disorders of the Teeth." In the course of his remarks he said he had been struck with the frequency with which carious teeth were associated with obstruction of the pharynx and enlarged tonsils; so much so that he made it a routine practice to examine the teeth in all cases of nasal obstruction, and he believed that there existed a relation between them; and he further is of opinion that there is a generic relation between some cases of vaulted arch, narrow jaws, and irregular teeth, and nasal obstruction. Normally we should breathe through the nose, so as to warm and filter the air respired. All animals, savage races, and young infants do so; but a large number of adults of civilized nations breathe through the mouth, because they have some obstruction of the nasal passages—erectile tumors, permanent catarrhal affections, polypi, post-nasal adenoid growths, etc. Mouth-breathing, he said, as a predisposing cause of caries of the teeth, came into action in various ways. The teeth were exposed to a current of air of a much lower temperature than that of the body, which would tend to cause inflammation of the periosteum and pulp of a tooth; the cold dry air produced congestion of the mucous membrane, with a secretion of stringy acid mucus; and the rapid evaporation of water which takes place when the mouth is constantly open inspissated this mucus, which so formed a fertile soil for the development of micro-organisms. Again, when sleeping with the mouth open, the tongue falls back and the parotid secretion finds its way directly through the pharynx instead of bathing and washing the teeth. With reference to the so-called V-shaped maxilla, Dr. Spicer thought that many cases might be traced to mouth-breathing, the muscles of the cheek pressing unduly upon the soft alveoli when the mouth is open.—*Lancet*, Jan. 8, 1890.

